# Hypoperfusion in nucleus accumbens in chronic migraine using 3D pseudo-continuous arterial spin labeling imaging MRI

**DOI:** 10.1186/s10194-022-01444-6

**Published:** 2022-06-27

**Authors:** Mengqi Liu, Yijie Sun, Xin Li, Zhiye Chen

**Affiliations:** 1 Department of Radiology, Hainan Hospital of Chinese PLA General Hospital, Sanya, 572013 China; 2 Department of Radiology, First Medical Center of Chinese PLA General Hospital, Beijing, 100853 China; 3 The Second School of Clinical Medicine, Southern Medical University, Guangzhou, 510515 China

**Keywords:** Arterial spin labelling, Brain, Chronic migraine, Magnetic resonance imaging, Nucleus accumbens, Perfusion imaging

## Abstract

**Background:**

Nucleus accumbens (NAcc) played an important role in pain mediation, and presents changes of neuronal plasticity and functional connectivity. However, less is known about altered perfusion of NAcc in chronic migraine (CM). The aim of this study is to investigate the altered perfusion of the NAcc in CM using a MR three-dimensional pseudo-continuous arterial spin labeling (3D PCASL) imaging.

**Methods:**

Thirteen CM patients and 15 normal controls (NC) were enrolled and underwent 3D PCASL and brain structural imaging. The cerebral blood flow (CBF) images were co-registered with the brain structural images, and the volume and CBF value of NAcc were extracted from the raw brain structural images and co-registered CBF images using an individual NAcc mask, which was obtained from the AAL3 template under transformation by the inverse deformation field generated from the segmentation of the brain structural images. The independent sample *t* test and receiver operating characteristic (ROC) curve was used to investigate the altered volume and perfusion of the NAcc in CM patients.

**Results:**

There was no significant difference for the volume of bilateral NAccs between CM and NC (*p* > 0.05). CM presented a lower CBF value (49.34 ± 6.09 ml/100 mg/min) compared with that of NC (55.83 ± 6.55 ml/100 mg/min) in left NAcc (*p* = 0.01), while right NAcc showed no significant difference between CM and NC (*p* = 0.11). ROC analysis identified that the area under the curve was 0.73 (95CI% 0.53–0.88) with cut-off value 48.63 ml/100 mg/min with sensitivity 50.00% and specificity 93.33%. The correlation analysis found a negative correlation between the CBF value of the left NAcc and VAS score (r = -0.61, *p* = 0.04).

**Conclusion:**

Hypoperfusion of the left NAcc was observed in CM, which could be considered as a potential diagnostic imaging biomarker in CM.

## Background

Migraine is a common primary headache with a reported prevalence of 9.3% of general population in China [1]. And it is also a main cause of chronic headache. It was reported that approximately 2.5% of chronic migraine (CM) was transformed from episodic migraine annually [2], and affected approximately 2% of the adult population in western countries [3] and 0.6–1.7% in Asia–Pacific region [4], which would generate substantial burdens on individual sufferers, their families and society. Despite the etiopathogenesis of migraine attributing it to cortical spreading depression (CSD) and activation of trigeminovascular system [5], the rigorous neuromechanism of migraine chronicization remains unknown.

Brain structural and resting-state functional MR imaging has been widely used to investigate the neuromechanism of CM, such as volume of hypothalamus as a diagnostic biomarker of chronic migraine [3], regional volume changes of the brain in migraine chronification [6], increased cerebral iron over the whole brain in chronic migraine [7, 8], and decreased functional connectivity of amygdala [1]. Above mentioned MR imaging techniques would partially reveal the neuromechanism of migraine chronification from the brain structural and functional aspects. However, the cerebral perfusion investigation was rarely reported in CM. Therefore, the aim of the current study was to investigate the cerebral perfusion change in CM using 3D pseudo-continuous arterial spin labelling (3D PCASL).

Nucleus accumbens (NAcc) is a subcortical brain structure which involves in pleasure, reward and addiction regulation. In the current viewpoints, NAcc also plays an important role in the pain mediation [9] that including: (1) brain structural level: projecting to and receiving information from key pain structures, such as the prefrontal cortex, anterior cingulate cortex, periaqueductal gray, thalamus, etc.; (2) brain function level: involving opioid regulation, motivation for avoiding pain and responding heavily to painful stimuli. In chronic pain, NAcc can present changes of neuronal plasticity and functional connectivity [10] and may signal pain-related events based on the decreased and increased dopamine signal [11]. However, the elaborate neuromechanism of NAcc in CM still remained unclear.

In the current study, we hypothesize that CM patients might present altered perfusion in NAcc. To address this hypothesis, we prospectively enrolled 13 CM patients and 15 normal controls (NC), and a 3D PCASL and brain structural imaging were performed in headache interictal period. An advanced segment technique was used to automatically measure the cerebral blood flow (CBF) value to elucidate the hypothesis.

## Methods

### Subjects

Thirteen CM patients were enrolled from headache clinic and 15 normal controls (NCs) from the hospital staffs or their relatives in Chinese PLA General Hospital. The inclusion criteria for chronic migraine patients should be fulfilled as follows: (1) CM refers to 1.3 in ICHD-III [12]: headache occurring on 15 or more days per month and lasts for more than 3 months, which has the features of migraine headache on at least 8 days per month and without aura; (2) absence of other subtypes of headache, chronic pain other than headaches, severe anxiety or depression, and psychiatric diseases; (3) without migraine preventive medication in the past 3 months; (4) absence of alcohol, nicotine, or other substance abuse; (5) no cerebral infarction, malacia or occupying lesions on the conventional MRI; (6) right-handed. NCs should never have had any primary headache disorders or other types of headaches in the past year. Inclusion criteria were similar to those of patients, except for the first items. The exclusion criteria for CM patients and NCs included as following: cranium trauma, chronic disorders such as hypertension, diabetes mellitus, and coronary heart disease etc. All the CM patients and NCs should have no MRI contraindications such as metal clips within the body and claustrophobia.

The clinical evaluations for the CM patients were as following: disease duration (DD), migraine frequency (MF), visual analogue scale (VAS) and migraine disability assessment (MIDAS) and standard categorical four-grade sleep disturbance scale (SDS) (0, normal; 1, mild sleep disturbance; 2, moderate sleep disturbance; 3, serious sleep disturbance). Written informed consents were obtained from all participants according to the approval of the ethics committee of the Chinese PLA General Hospital.

### MR imaging

A GE 3.0 T MR scanner (DISCOVERY MR750, GE Healthcare, Milwaukee, WI, USA) and a conventional eight-channel quadrature head coil was used to acquire the MR images. The brain structural data were obtained from an axial three-dimensional T1-weighted fast spoiled gradient recalled echo (3D T1-FSPGR) sequence with slices number = 360, repetition time (TR) = 7.0 ms, echo time (TE) = 3.0 ms, flip angle = 15 ^o^, field of view (FOV) = 25.6 cm × 25.6 cm, Matrix = 256 × 256, slice thickness = 1 mm, number of acquisitions = 1. The brain perfusion data were obtained by using an axial pseudo-continuous arterial spinal labelling (PCASL) tagging scheme with a 3D interleaved spiral fast spinal echo (FSE) readout (3D spiral FSE ASL): TR/TE = 5128 ms/15.9 ms, flip angle = 111°, FOV = 20 cm × 20 cm, x, y matrix = 1024 × 8 (spiral acquisition), slice thickness = 3.0 mm, labeling duration was 1.5 s, and post-labeling delay time (PLD) was 1.5 s. After PCASL images acquisition, MR scanner would automatically generate 50 slices of CBF maps by using Functional tools (version:9.4.05) [13].

The conventional MR imaging included as following: oblique T2 weighted imaging (T2WI), diffusion weighted imaging (DWI) and T2 fluid attenuated inversion recovery (T2-FLAIR). These images were used to exclude the subjects with obvious brain lesions. All the subjects underwent the same MR protocols and were scanned by the same MR technician.

All MRI examinations were performed in the headache interictal period for the CM patients, and the alcohol, nicotine, caffeine and other substances were avoided at least 12 h before MRI examination.

### Image processing

**Fig. 1:**
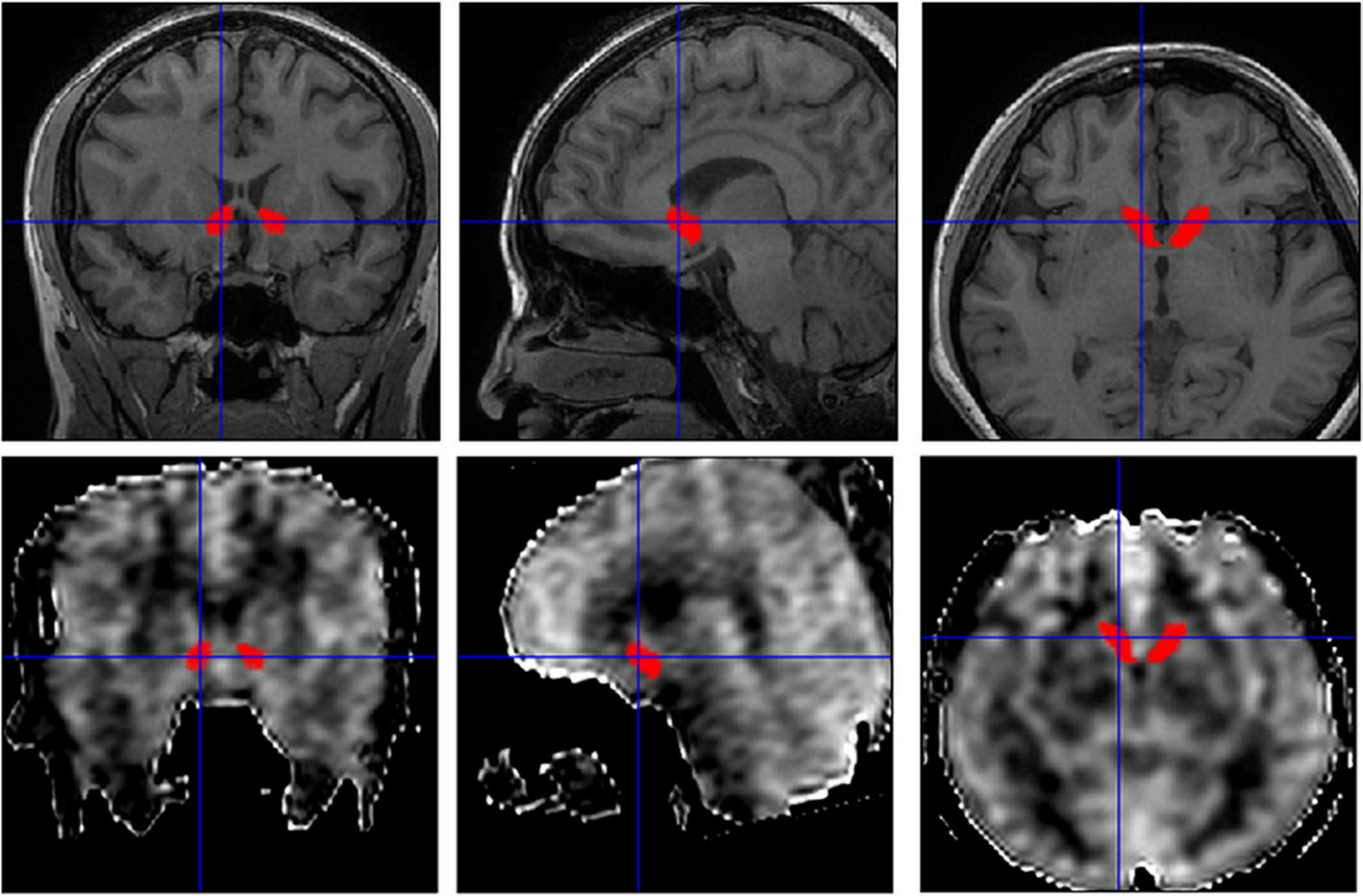
The individual nucleus accumbens segment. Top line, raw brain structural images; bottom line, raw CBF images co-registered with the brain structural images. Red masks, individual bilateral nucleus accumbenses by applied with inverse deformation field

Statistical Parametric Mapping 12 (SPM 12) and CAT12 plugin (http://www.fil.ion.ucl.ac.uk/spm/) were used to preprocess the MR image under MATLAB 7.6 (The Mathworks, Natick, MA, USA). The image processing included following steps: (1) The structural image (3D T1-FSPGR) were segmented by using CAT12 to generate inverse deformation field (IDF); (2) The IDF was applied to NAcc template obtained from AAL3(v1.0) [14], and this would generate individual NAcc mask (Fig. 1); (3) The CBF images were co-registered with raw T1 image (3D) and generated co-registered CBF image (corCBF); (4) All the individual NAcc masks were checked by overlaying the individual mask to corCBF images to identify the quality of the CBF images, and then one patient was excluded due to the artifact of anterior skull base. (5) The CBF value of NAcc was extracted from the corCBF based on the individual NAcc mask.

### Statistical analysis

The data with normal distribution presented as mean ± standard deviation, and the data with non-normal distribution presented by median (minimum, maximum). The quantitative variables were performed with independent sample *t* test, and the qualitative variable gender were performed with Chi-Square test. The variable CBF of NAcc was performed with Jonckheere-Terpstra trend test. The Pearson correlation was performed with the data with normal distribution, and the Spearman correlation was performed with the data with non-normal distribution and qualitative data. Significant difference was set to *p* value < 0.05. The statistical analysis was performed by using MedCalc(V19.0.4).

Receiver operating characteristic (ROC) curve analysis was performed to identify the diagnostic efficacy of the perfusion of NAcc for CM, and area under the curve (AUC) was recognized reasonable diagnostic valuable with AUC set at > 0.7.

## Results

The current study included 13 CM patients and 15 NCs. The age and gender showed no significant difference between two groups (*p* value was 0.44 and 0.1, respectively).

### Comparison of the volume and perfusion of NAcc between CM and NC

**Fig. 2:**
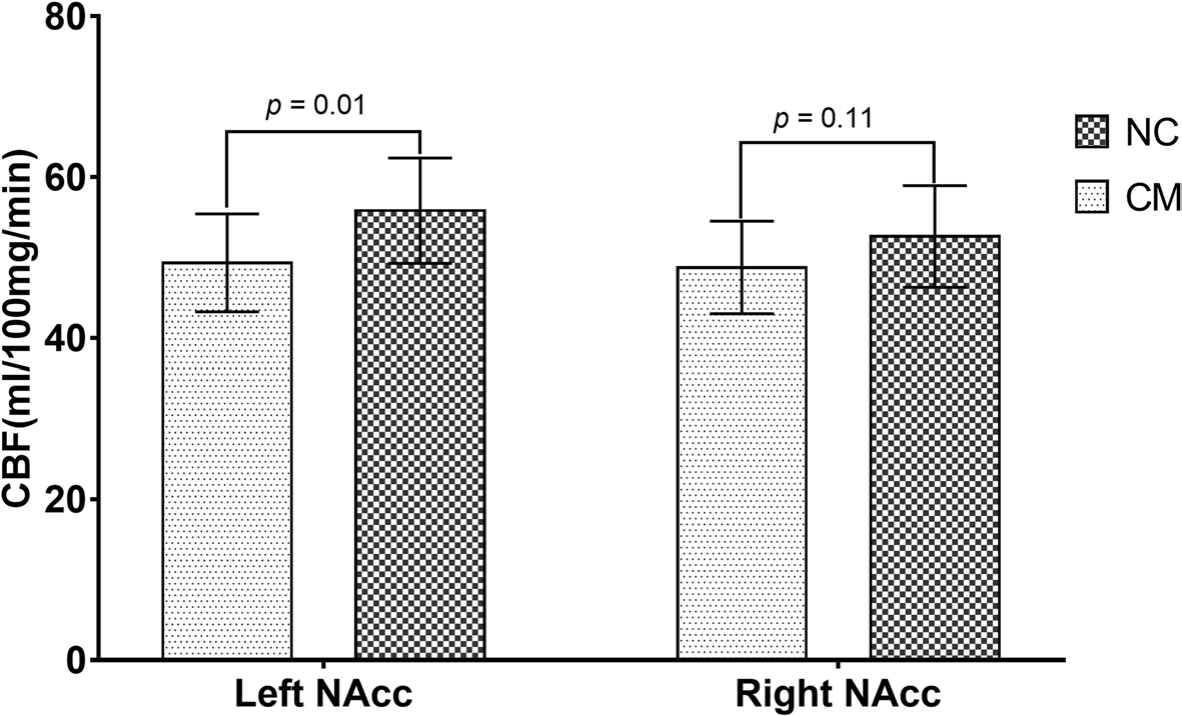
The CBF value of bilateral nucleus accumbenses in CM and NC. The left NAcc presented significant hypoperfusion (*p* = 0.01), and the right NAcc showed no significant hypoperfusion (*p* = 0.11). NAcc, nucleus accumbens; CM, chronic migraine; NC, normal control; CBF, cerebral blood flow

The perfusion of left NAcc reduced significantly in CM (49.34 ± 6.09 ml/100 mg/min) compared with NC (55.83 ± 6.55 ml/100 mg/min) (*p* = 0.01), while the perfusion of right NAcc showed no significant difference between CM (48.76 ± 5.75 ml/100 mg/min) and NC (52.62 ± 6.33 ml/100 mg/min) (*p* = 0.11) (Table 1 and Fig. 2). Further Jonckheere-Terpstra trend test presented that there was no significant decreased trend for the CBF value of right NAcc in CM compared with NC (Z value = -1.37, *p* = 0.17).

**Table 1:** Comparison of the volume and perfusion of NAcc between CM and NC

	Left NAcc		Right NAcc	
	Volume(ml)	Perfusion^a^	Volume(ml)	Perfusion^a^
CM	0.40 ± 0.04	49.34 ± 6.09	0.31 ± 0.03	48.76 ± 5.75
NC	0.40 ± 0.04	55.83 ± 6.55	0.31 ± 0.04	52.62 ± 6.33
*t* value	0.07	2.64	0.22	1.64
*p* value	0.94	0.01	0.83	0.11

^a^ml/100 mg·min

Table 1 presented that there was no significant difference for volume of NAcc between CM (left, 0.40 ± 0.04 ml; right, 0.40 ± 0.04 ml) and NC (left, 0.31 ± 0.03 ml; right, 0.31 ± 0.04 ml) (*p* > 0.05).

### ROC analysis of the CBF value of the left NAcc in CM

**Fig. 3:**
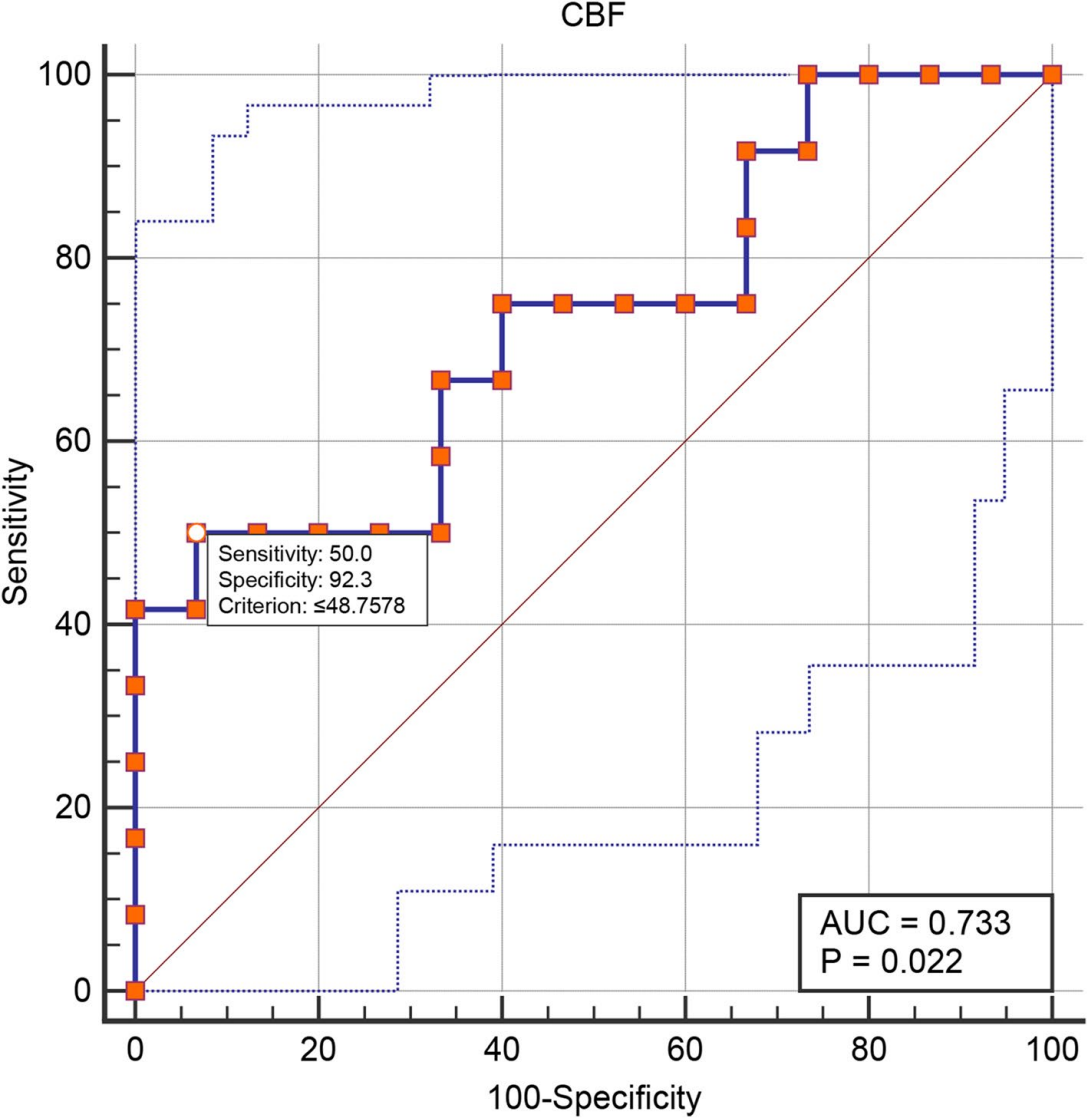
ROC curve for the CBF value of the left NAcc to diagnose CM from NC. The area under the curve was 0.73 with the cut-off value was 48.76 ml/100 mg/min

**Fig. 4:**
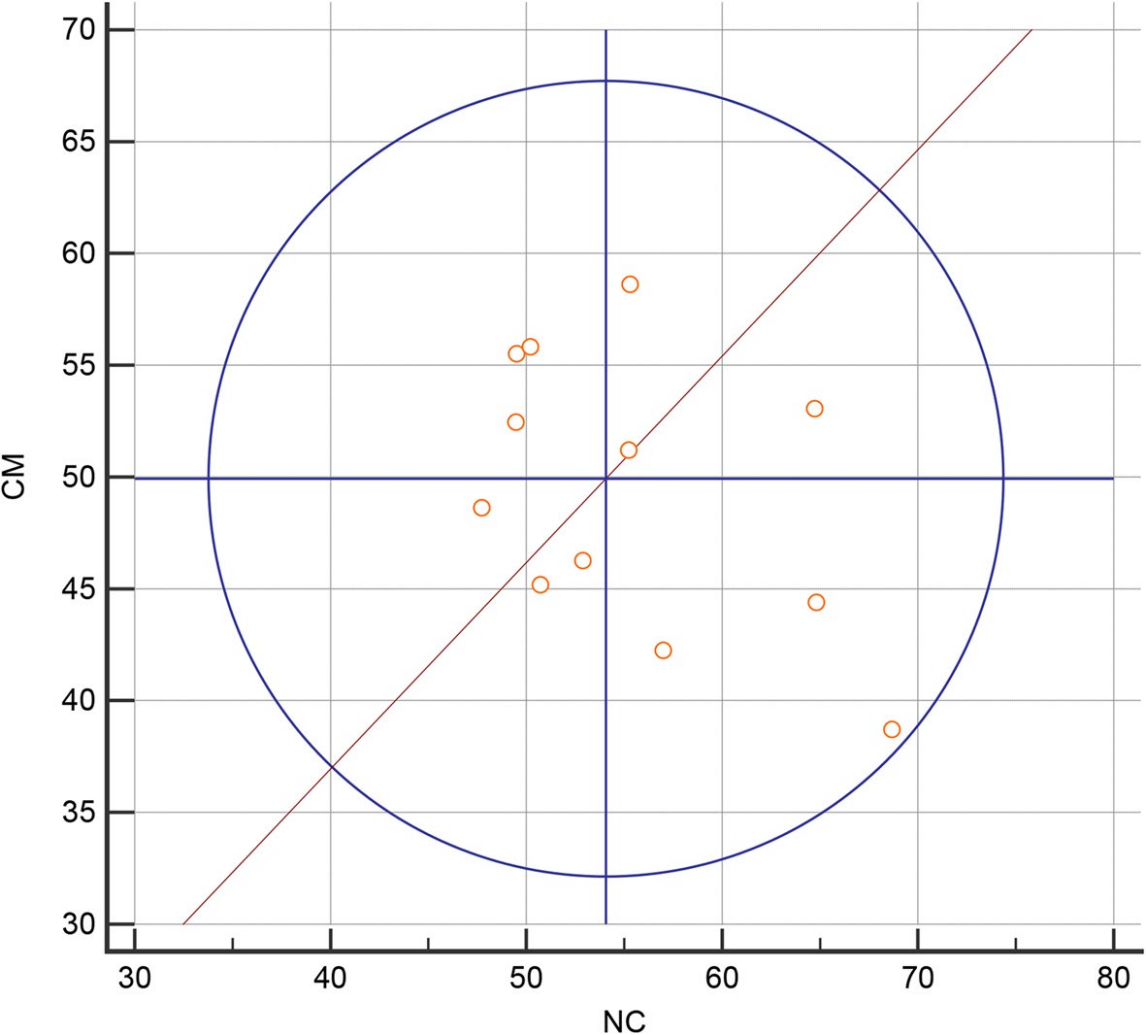
Youden plot for the CBF value of CM and NC, which indicated a small total error with all the points in the circle. The circle represented the 95% coverage probability, and all the point in the circle with a small total error

Figure 3 presented that the area under ROC curve for the CBF value of the left NAcc was 0.73 (95CI% 0.53–0.88), and the cut-off value is 48.63 ml/100 mg/min with sensitivity 50.00% and specificity 93.33% in distinguishing CM from NC. The Youden plot indicated a small total error with all the points in the circle (Fig. 4).

### Correlation analysis of the CBF value of the left NAcc with the clinical variables in CM

There was a negative correlation between the CBF value of the left NAcc and VAS (r = -0.61, *p* = 0.04). Other clinical variables (MIDAS, disease duration, migraine frequency and sleep disturbance scale) presented no significant correlation with the CBF value of the left NAcc (*p* > 0.05) (Table 2).

**Table 2:** The correlation analysis of the CBF value of the left NAcc with the clinical variables

Varibale	Mean	r	*p* value
VAS	7.92 ± 1.31	-0.61	0.04
MIDAS	105.33 ± 48.57	0.08	0.81
DD(years)	9.00(3.00,30.00)	0.05	0.87
MF	30(17,30)	0.13	0.70
SDS	3(0,4)	-0.05	0.87

*VAS* Visual Analog Scale, *MIDAS* Migraine Disability Assessment Scale, *DD* Disease duration, *MF* Migraine frequency, *SDS* Sleep disturbance scale

## Discussion

NAcc is centered in the limbic circuit of basal ganglia, and provided an interface between the limbic and the motor system [15]. It participate in the emotion control, decision-making [16] and mediating motivational processes [17]. As a part of striatum, NAcc was not commonly related with pain from the conventional points of view. However, some previous studies have reported that NAcc was associated with the chronic pain [18, 19]. The current study also demonstrated that NAcc might be another target brain regions that worth to be paid for more attention in CM.

Chronic migraine may present the altered cerebral volume changes, which had been confirmed decreased volume of hypothalamus [3], increased volume of right putamen, left lingual gyrus, and left occipital fusiform gyrus, decreased volume of left basal forebrain [6]. However, a previous study demonstrated that there were no significant volume changes over the whole brain in CM using a voxel-based morphometry (VBM) [20]. Therefore, the automatic ROI method was used to extract the volume and perfusion CBF value of NAcc in CM in the current study. The current study demonstrated that there were no significant volume changes of the bilateral NAccs in CM compared with NC, which suggested that the structural impairment of NAcc could not be observed in CM, and the further functional evaluation of NAcc might provide more valuable information to understand the neural mechanism of CM. These findings also indicated that NAcc could not presented volume changes in top-down modulation of pain [21]. It was known that NAcc and its connections could provide a common circuit for drug addiction, and a previous study demonstrated that heroin addiction could induced a reduced volume of NAcc. Therefore, the altered volume of NAcc might offer insights into disease-related changes in vivo from CM to medication-overuse headache [22].

Modern neuroimaging techniques could provide much more information to help to reveal the neuromechanisms of CM [3, 7, 23]. The current study identified that the left NAcc presented hypoperfusion in CM, which had high specificity for the diagnosis of CM. Otherwise, our previous study demonstrated that the increased CBF value of the left Brodmann 38 in episodic migraine [13]. Therefore, these findings suggested that altered perfusion of the left NAcc might involve in the migraine chronicization. The hypoperfusion of NAcc might also be related with the higher functional connectivity of the NAcc with the ventromedial prefrontal cortex, which predicted transition from subacute to chronic pain [24] and in part reflected a compensatory mechanism as activation of the medial prefrontal cortex–NAcc pathway [25].

In the current study, right NAcc presented no significant decreased CBF value in CM compared with NC by using independent sample t test, and further Jonckheere-Terpstra trend test also did not revealed the significant decreased trend of the CBF value in right NAcc in CM compared with NC. Although the significant positive results were not detected in right NAcc in CM, this pilot study’s findings should not be interpreted as there was no any change for the perfusion state of right NAcc in CM. However, some factors might prevent subtle altered perfusion change in right NAcc in CM in the current study, such as small sample size, preprocessing methods, PCASL technique, and etc. One previous study [26] demonstrated that the placement of electrode in right NAcc could improve the left hemibody pain rating in a patient with central poststroke pain, which might reveal that NAcc might have the lateralization predilection in the pain processing. The other MRI study [27] investigated that the pain intensity in the "off" state and from the "on" to "off" state were both substantially correlated with the functional connectivity (FC) changes between the NAc and contralateral NAc, which also suggested that bilateral NAcc contributed the pain processing in pain-related abnormal neuronal synchronization in Parkinson's disease. Therefore, bilateral NAcc should deserve attention in the future pain study.

The VAS was commonly used to evaluate the levels of headache in clinical practice, and the current study confirmed that the negative correlation presented in the perfusion of left NAcc and VAS score in CM during migraine interictal period. The altered interictal perfusion may reflect local interictal differences in neuronal metabolism or activity, or the presence of some degree of interictal cerebrovascular dysregulation in migraineurs [28]. A previous study also confirmed that the extent of cerebral perfusion could negatively predicted the pain intensity [29]. Therefore, the noninvasive quantitative CBF measurement with advanced segment techniques could be considered as an optimal strategy for the objective evaluation of the painful information in the CM.

This study had some limitations. First, the sample size was not large enough for the clinical relevance analysis, e.g. headache laterality, headache-free time, different phase of a migraine attack and post-attack, etc. The correlation would be investigated between these clinical parameters and altered perfusion of NAcc. Therefore, the current study could only be considered as a pilot investigation. Second, the current study only involved brain structural and perfusion, and it would need further other functional MRI to investigate the plasticity in NAcc circuit. Third, the cerebral perfusion of the whole NAcc was measured, and the subregions of NAcc, including NAcc shell and NAcc core, were not segmented and performed with CBF extraction.

## Conclusions

In conclusion, the hypoperfusion of the left NAcc was observed in CM, and could be considered as an imaging biomarker for the objective evaluation of chronic painful information in CM. Noninvasive brain perfusion imaging might provide novel insights into the central mechanisms of CM.

## Data Availability

All the data supporting our findings is contained within the manuscript.

## Abbreviations

AUC: Area under the curve
CBF: Cerebral blood flow
CM: Chronic migraine
MF: Migraine frequency
MIDAS: Migraine Disability Assessment Scale
NAcc: Nucleus accumbens
NC: Normal control
PCASL: Pseudo-continue arterial spin labelling
ROC: Receiver operating characteristic curve
SDS: Sleep disturbance scale
VAS: Visual Analog Scale
